# Nitrous Oxide

**Published:** 1881-11

**Authors:** 


### EDITORIAL.

### NITROUS OXIDE.

A contributor to the Dental News of October, says: "One of the popular agents of this day is Nitrous Oxide; and I recognize in it all the good properties claimed for it, but then we all know it deteriorates. So few are prepared to manufacture their own gas, and even if they are, it is very difficult to make it pure. But granting that it is pure originally, it cannot be kept for any great length of time. There is no kind of receiver made that will keep it absolutely pure. And after it deteriorates, it is extremely dangerous." By the side of the above quotation, I wish to place the following:

Ten years ago, Dr. S. P. M., of a city in Massachusetts, said to me in a conversation upon nitrous oxide, that about six months before he had filled his gas holder, a zinc one over a tank of water, he was then from his office four months, when he returned, he found the gas there. Being desirous to know whether there had been any change in the gas, he first inhaled it himself, to entire anæsthesia, and then gave it to others till it was all used, and no change in the slightest degree was recognized in it, neither in its taste or its action; it produced anæsthesia as promptly and as perfectly as though it had been prepared but a few hours before. Did it deteriorate, or was it *extremely dangerous* in that instance? Dr. W. of the Dental College of the University of Michigan, about the first of July last, filled his gas holder, and it remained undisturbed till about the middle of September, when it was used, and found to be in every respect in a perfect state, no appreciable change was apparent by any ordinary test. It produced anæsthesia with promptness and efficiency. Was this also *extremely dangerous*? In each of these cases the gas was made in the office.

Seven years ago, a bottle of liquid nitrous oxide was sent to the Dental College of the University of Michigan. A part of the gas was used within three or four months after it was received, the bottle was then put aside; within the last few days it has been brought forth, and part of the seven year old gas used, and in every particular, it seemed as perfect as though made but yesterday, producing entire anæsthesia in a few moments, without the slightest unfavorable symptom. Was this too extremely dangerous? Will M. E. S. please tell us in what the deterioration, to which he refers, consists, and how it is effected? Does a new compound result from the deterioration? If so, what is it? What influences produce the deterioration? It is to be hoped that M. E. S. will give a reason for the faith that is in him.

